# 非小细胞肺癌患者术后血清VEGF动态变化与血小板的相关性研究

**DOI:** 10.3779/j.issn.1009-3419.2010.02.07

**Published:** 2010-02-20

**Authors:** 瑛 胡, 宝兰 李, 广利 时, 长利 荣, 广阔 高

**Affiliations:** 101149 北京，北京胸科医院综合科 Department of General, Beijing Chest Hospital, Beijng 101149, China

**Keywords:** 肺肿瘤, 血管内皮生长因子, 酶联免疫吸附试验, 手术, 血小板, Lung neoplasms, Vascular endothelial growth factor (VEGF), Enzyme linked immunosorbent assay (ELISA), Surgery, Platelet

## Abstract

**背景与目的:**

已有研究表明:非小细胞肺癌(non-small cell lung cancer, NSCLC)患者手术切除原发肿瘤后其血清中血管内皮生长因子(vascular endothelial growth factor, VEGF)浓度显著升高, 血小板可能是血清中VEGF的主要来源。本研究的目的是探讨NSCLC患者术后血清VEGF浓度的动态变化及其与血小板之间的关系。

**方法:**

应用酶联免疫吸附试验(enzyme linked immunosorbent assay, ELISA)检测法, 监测76例非小细胞肺癌患者术前、术后1天及7天血清VEGF的浓度, 同期检测血小板的浓度。

**结果:**

① NSCLC患者术前、术后1天及7天血清VEGF分别为(842.06±527.24)pg/mL、(1 119.28±609.62)pg/mL、(1 574.09±873.38)pg/mL, 组间比较差异具有统计学意义(*P* < 0.001);②NSCLC患者术前、术后1天及7天血小板计数分别为(230.42±82.56)×10^9^/L、(196.47±81.48)×10^9^/L、(237.90±86.94)×10^9^/L, 术后1天最低(*P* < 0.001);③术后7天在血小板高于均数组血清VEGF浓度为(1 842.86±1 006.63)pg/mL, 低于均数组为(1 398.81±734.00)pg/mL, 两组有统计学差异(*P*=0.043)。

**结论:**

NSCLC患者术后血清VEGF浓度显著升高, 血小板计数高的患者中, 其血清VEGF浓度升高更为明显。

## Abstract (en)

**Background and objective:**

It ha been proven that serum vascular endothelial growth factor (VEGF) concentration was elevated significantly after surgery in patients of non-small cell lung cancer (NSCLC).Platelet may be the main resource of serum VEGF.The aim of this study is to investigate the correlation between postoperative dynamic changes of serum VEGF levels and platelet counts in patients of NSCLC who underwent surgery.

**Methods:**

Serum VEGF levels were determined in 76 patients of NSCLC who were treated with surgery by ELISA(enzyme linked immunosorbent assay) method before operation and on postoperative day 1, 7.At the same day the concentrations of platelet were determined.

**Results:**

① Serum VEGF in patients of NSCLC on preoperative day, postoperative 1 day and 7 day were (842.06±527.24)pg/mL, (1 119.28±609.62)pg/mL, (1 574.09±873.38)pg/mL, respectively (*P* < 0.001);②Platelet counts in patients of NSCLC on preoperative day, postoperative 1 day and 7 day were (230.42±82.56×10^9^/L, (196.47±81.48)×10^9^/L, (237.90±86.94)×10^9^/L; the value on postoperative 1 day was the lowest (*P* < 0.001);③On postoperative 7 day, serum VEGF in the group of lower than the mean and higher than the mean were respectively (1 398.81±734.00)pg/mL and (1 842.86±1 006.63)pg/mL (*P*=0.043).

**Conclusion:**

Serum VEGF in patients of NSCLC after surgery were elevated.In the group of higher platelet counts, serum VEGF increased more significantly.

肿瘤的生长、侵袭和转移有赖于血管生成(angiogenesis)^[1, 2]^。许多血管生成因子参与了肿瘤的血管生成过程^[3, 4]^。其中, 血管内皮生长因子(vascular endothelial growth factor, VEGF)在肿瘤的血管生成中起重要作用^[5-7]^。许多研究^[8-12]^证实非小细胞肺癌(non-small cell lung cancer, NSCLC)患者血清VEGF水平显著升高。近年有报道^[13-15]^NSCLC患者手术切除原发肿瘤后其血清中VEGF显著升高。VEGF储存于血小板膜的α-质粒中^[16]^, 在凝血过程中由于血小板活化而被释放^[17]^。研究^[18]^发现肿瘤患者血小板计数与血清VEGF水平呈正相关, 有作者^[19-21]^提出血小板可能是血清中VEGF的主要来源。尚不清楚NSCLC患者手术前后血小板的动态变化情况。因此本文目的是通过监测NSCLC患者手术前后血清VEGF浓度及血小板计数动态变化, 分析两者之间是否有相关性。

## 1 材料与方法

### 1.1 研究对象

76例2008年5月-2008年10月于本院胸外科行肺切除手术的NSCLC患者, 监测行肺切除手术前后患者血清VEGF浓度及血小板计数的动态变化。患者的入选标准:①于术前经细胞学或病理学确诊, 或经手术病理证实的Ⅰa-Ⅲb期NSCLC患者; ②术前常规检查及功能评价均符合手术的适应证; ③均未经化疗、放疗或其它与抗肿瘤相关的治疗; ④近两周内无外伤或其它手术治疗史; ⑤无视网膜病变; ⑥无缺血性心脏疾病; ⑦年龄 > 18岁; ⑧女性患者非月经期。

### 1.2 临床资料

按照UICC(1997)分期标准进行术后病理分期。中位年龄为58.05岁(33岁-79岁); 男性65例, 女性11例; 腺癌34例, 鳞癌34例, 腺鳞癌8例; 低分化7例, 中分化69例; Ⅰ期35例(Ⅰa期10例, Ⅰb期25例)、Ⅱ期10例(Ⅱa期1例, Ⅱb期9例)、Ⅲ期31例(Ⅲa期29例, Ⅲb期2例)。

### 1.3 主要仪器及试剂

人类VEGF检测试剂盒(R&D有限公司), Thermo labsysstems酶标仪(芬兰), BioRAD MODEL1575洗板机(芬兰), Eppendrop低温高速离心机(德国), 37 ℃ DHP120型温孵箱(上海市实验仪器总厂), 血球计数仪1800i(日本)。

### 1.4 标本的采集及检测

分别在手术前1天-2天、术后1、7天, 清晨空腹抽取患者外周静脉血, 用于检测VEGF的血标本不抗凝, 取血后当日离心(1 000 rpm)10 min, 分离血清, 置于-80 ℃保存待测定; 同日非抗凝管封装血标本后2 h内检测血小板。采用酶联免疫吸附试验法(enzyme linked immunosorbent assay, ELISA)测定血清VEGF浓度。

### 1.5 统计学分析

结果以Mean±SD表示, 应用SPSS 13.0统计软件包处理, 组间比较应用*t*检验、方差分析, 相关性采用*Pearson*相关分析, *P* < 0.05为差异具有统计学意义。

## 2 结果

### 2.1 围术期血清VEGF的变化

NSCLC患者术前、术后1天、术后7天的血清VEGF分别为(842.06±527.24)pg/mL、(1 119.28±609.62)pg/ mL及(1 574.09±873.38)pg/mL, 三者比较差异具有统计学意义(*F*=22.05, *P* < 0.001), 其中术前与术后1天(*t*=-4.634, *P* < 0.001)、术后7天与术前(*t*=-10.192, *P* < 0.001)及术后1天与术后7天(*t*=-6.092, *P* < 0.001)比较均有统计学差异(图 1)。

**1:**
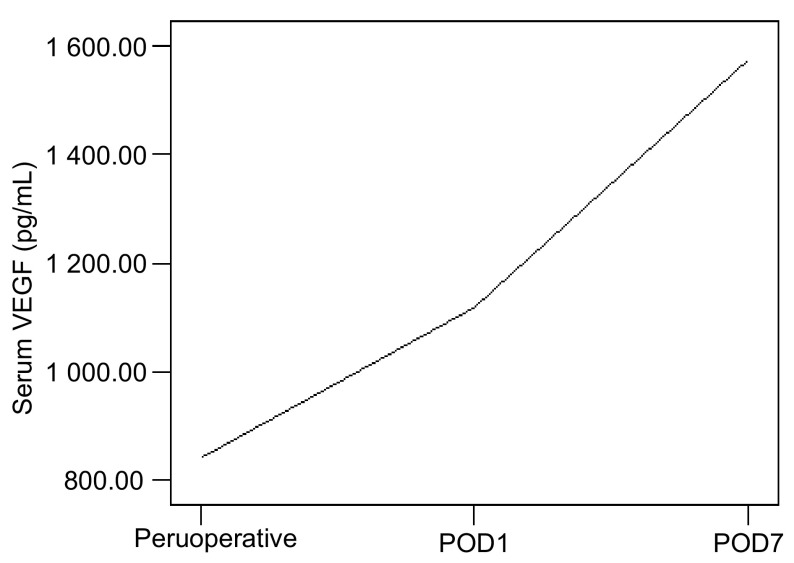
NSCLC患者围术期血清VEGF的变化 Peruoperative danymic changes of serum VEGF in patients of NSCLC

### 2.2 围术期血小板计数的变化

NSCLC患者术前、术后1天、术后7天的血小板计数分别为(230.42±82.56)×10^9^/L、(196.47±81.48)×10^9^/L及(237.90±86.94)×10^9^/L, 三者比较差异具有统计学意义(*F*=5.288, *P*=0.006), 其中术前及术后1天比较有统计学差异(*t*=4.309, *P* < 0.001);术后7天与术前比较无统计学差异(*t*=-0.521, *P*=0.353);术后1天及术后7天比较有统计学差异(*t*=-4.555, *P* < 0.001)(图 2)。

**2:**
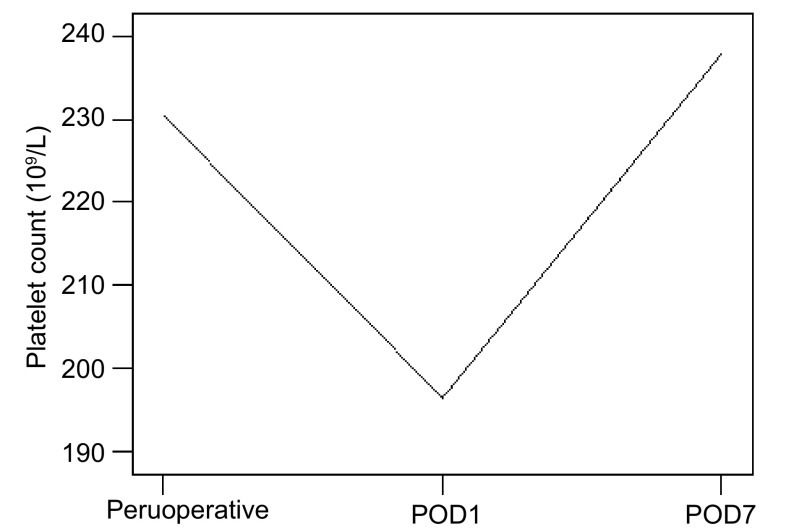
NSCLC患者围术期血小板计数的变化 Peruoperative danymic changes of platelet count in patients of NSCLC

### 2.3 围术期血小板计数与血清VEGF动态变化的相关性分析

NSCLC患者术前、术后1天及术后7天血清VEGF及血小板计数之间行相关性分析, 结果显示术前、术后1天及术后7天血清VEGF及血小板计数之间的相关系数分别为-0.069(*P*=0.554)、-0.093(*P*=0.424)及0.293(*P*=0.010), 其中术后7天两组间有显著相关性。分别以术前、术后1天及术后7天的血小板均数为分界值, 将所研究病例分为两组, 行统计学处理, 比较不同血小板水平的组间血清VEGF浓度, 术前(t=-0.349, *P*=0.728)及术后1天(*t*=1.181, *P*=0.242)之间比较无统计学差异, 术后7天两组间比较差异具有统计学意义(*t*=-2.223, *P*=0.029)(图 3)。

**3:**
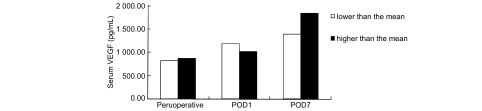
NSCLC患者血清VEGF浓度的组间比较(以血小板均数为分界值分组) Comparison of serum VEGF concentration among groups of NSCLC patients (grouped by the mean of platelet count)

## 3 讨论

VEGF即血管内皮通透因子(vascular endothelial permeability factor, VPF), 是血小板源生长因子(platelet devived growth factor, PDGF)家族的一个成员, 可由正常细胞和肿瘤细胞产生和分泌^[22]^。VEGF是一类糖蛋白, 广泛分布于人和动物体内的脑、肾、肝、脾、肺、骨骼等组织。VEGF是通过与其内皮细胞膜上的受体(VEGFR)结合发挥作用。VEGF对血管内皮细胞的增殖、水解基底膜、细胞迁移和血管构建的调控作用最强, 特异性最高^[23, 24]^。通过增加内皮细胞有丝分裂及迁移, 重塑细胞外基质及增加血管通透性, 从而调节病理性血管生成, 通过激活蛋白水解酶降解基质, 从基因水平上调多种蛋白酶及蛋白酶的激活物^[25]^。

研究^[13-15]^发现手术切除原发肿瘤后其血清中VEGF显著升高, 那么切除原发肿瘤后, VEGF主要来源于何处？1988年报道57%的小细胞肺癌患者中β-凝血球蛋白升高, β-凝血球蛋白本是血小板活化的标志, 其后发现NSCLC患者中β-凝血球蛋白水平同样显著升高^[26]^。有研究^[19-21]^提出血小板可能是血清中VEGF的主要来源。如果术后血小板是血清VEGF的主要来源, 那么术后两者水平之间必定呈正相关, 本文结果并不完全支持此推断。本文中再次证实血清VEGF水平于手术后明显升高, 术后1天及术后7天均呈显著上升趋势。血小板计数的动态变化与血清VEGF不同, 术后1天显著降低, 术后7天升至术前水平。目前尚无肺癌患者在手术前后血小板计数变化的相关报道。George^[18]^的研究表明, 结肠癌患者术后20 h内血小板及血清VEGF均显著升高, 且两者有显著相关性。术后1天血小板计数显著降低的原因尚待进一步研究, 但同一时间血清VEGF仍显著升高, 说明术后血清VEGF升高不仅来自血小板活化后的释放, 尚有其它组织或细胞释放VEGF。

本文中术前及术后1天的血清VEGF水平与血小板计数间无相关性, 术后7天两者有相关性, 进一步分析发现术后7天时, 血小板计数低于均数的组中, 血清VEGF水平显著低于血小板计数高于均数组的血清VEGF水平, 两组之间差异有统计学意义。说明血小板与VEGF之间可能有相互作用。凝血过程使血小板活化, 诱导了VEGF等多种肿瘤血管生成因子的释放^[27]^; 同时VEGF可以增加血小板的粘附能力, 促进凝血的发生, 从而诱导了血小板的活化^[28]^。有研究^[18]^认为随着VEGF升高, 血小板水平升高可能是血小板在发挥清除体内循环中VEGF的作用。

术后7天时血小板计数与术前水平相同, 但血清VEGF水平却升高显著。其原因可能为:①术后有其它合成及释放VEGF的途径, 有研究发现中性粒细胞中富含VEGF^[29, 30]^; ②手术激活了凝血机制使血小板活化, 从而术后血小板释放VEGF能力比术前显著提高。

综上所述, 术后血清VEGF水平显著升高, 血小板计数的动态变化与血清VEGF之间无正相关性, 但术后7天血小板计数高的组别中血清VEGF水平显著升高。尚待进一步了解血小板在肿瘤生长及转移中的作用, 更多了解血小板与肿瘤血管生成因子之间的关系, 从而为制定肺癌治疗方案提供更多的参考。
